# Protein- and Calcium-Mediated GLP-1 Secretion: A Narrative Review

**DOI:** 10.1093/advances/nmab078

**Published:** 2021-06-30

**Authors:** Jonathan D Watkins, Françoise Koumanov, Javier T Gonzalez

**Affiliations:** Centre for Nutrition, Exercise and Metabolism, Department for Health, University of Bath, Bath, United Kingdom; Centre for Nutrition, Exercise and Metabolism, Department for Health, University of Bath, Bath, United Kingdom; Centre for Nutrition, Exercise and Metabolism, Department for Health, University of Bath, Bath, United Kingdom

**Keywords:** protein, calcium, GLP-1, extracellular calcium-sensing receptor, metabolic regulation, type 2 diabetes

## Abstract

Glucagon-like peptide 1 (GLP-1) is an incretin hormone produced in the intestine that is secreted in response to nutrient exposure. GLP-1 potentiates glucose-dependent insulin secretion from the pancreatic β cells and promotes satiety. These important actions on glucose metabolism and appetite have led to widespread interest in GLP-1 receptor agonism. Typically, this involves pharmacological GLP-1 mimetics or targeted inhibition of dipeptidyl peptidase-IV, the enzyme responsible for GLP-1 degradation. However, nutritional strategies provide a widely available, cost-effective alternative to pharmacological strategies for enhancing hormone release. Recent advances in nutritional research have implicated the combined ingestion of protein and calcium with enhanced endogenous GLP-1 release, which is likely due to activation of receptors with high affinity and/or sensitivity for amino acids and calcium. Specifically targeting these receptors could enhance gut hormone secretion, thus providing a new therapeutic option. This narrative review provides an overview of the latest research on protein- and calcium-mediated GLP-1 release with an emphasis on human data, and a perspective on potential mechanisms that link potent GLP-1 release to the co-ingestion of protein and calcium. In light of these recent findings, potential future research directions are also presented.

## Teaser

**Statement of Significance**: Emerging evidence suggests the co-ingestion of protein and calcium can potently stimulate GLP-1 release.

## Introduction

The obesity and type 2 diabetes (T2D) epidemics are major health concerns, whereby 13% and ∼6.3% of the global adult population (18 y or over) had obesity (1) and T2D (2) in 2016 and 2014, respectively. Furthermore, T2D is predicted to cost the UK National Health Service ∼£17 billion per year by 2035 (3), and obesity already costs >$2 trillion per year, globally (4) The current most effective long-term approach to treating obesity is bariatric surgery (5). Roux-en-Y gastric bypass surgery (RYGB) produces ∼63% excess body weight loss (difference between initial and final BMI) after 2 y (6), and T2D remission rates of >40% at 12 mo and at the time of latest follow-up (median, 23 mo) (7). Despite this, surgery is unlikely to be economically viable at the population level due to its cost of ≤£61,000 per quality-adjusted life year (8). Nevertheless, by establishing the mechanisms by which surgery can enhance weight loss and remission from diabetes, potential surgery “mimetics” can be explored to harness the weight-loss benefits of surgery, without the costs or the need for invasive procedures. A reported key mechanism for the surgery-induced weight loss and diabetes remission is greater nutrient delivery to intestinal epithelial L-cells and the associated enhancement of gut hormone availability (9–12). This suggests that targeting gut hormones released from intestinal epithelial cells holds promise as an obesity/T2D therapy.

Within the intestinal epithelium, enteroendocrine cells represent <1% of all gastrointestinal (GI) cells, and are a key component of the gut-brain-pancreas axis (13, 14). These enteroendocrine cells are categorized into a number of classes depending on their location along the GI tract, receptor expression, and secretory profile (15). Glucagon-like peptide 1 (GLP-1) is a peptide hormone produced and secreted by intestinal L-cells, which are located along the length of the GI epithelium, starting at the jejunum and then increasing in density along the small and large intestine down to the colon (10). GLP-1 has received a lot of attention because it is classified as an incretin hormone (hormones that stimulate glucose-dependent insulin secretion from the pancreas) along with glucose-dependent insulinotropic polypeptide (GIP). Additionally, GLP-1 also regulates appetite in a similar manner to hormones such as peptide tyrosine-tyrosine (PYY) and leptin, to promote fullness and reduce energy intake (16, 17). These collective actions have led to widespread interest in GLP-1 as a potential target for obesity and diabetic management and therapy.

Subsequently, agonists for the GLP-1 receptor have been a popular therapeutic option. Clinical trials that have involved GLP-1 receptor agonists such as exenatide and liraglutide have led to weight loss of ≤3 kg, and improvements in glycated hemoglobin of 2%, over 15–30 wk (18). More recently, 2.4 mg subcutaneous semaglutide administered once weekly for 68 wk (alongside a lifestyle intervention) resulted in 14.9% weight loss from baseline in obese individuals (19). Despite this, using a nutritional intervention to target L-cells and endogenous gut hormone release is still relevant as an alternative or combined treatment, due to a number of possible advantages over GLP-1 agonists. Firstly, nutritional intervention minimizes the potential adverse side effects, including GI discomfort and nausea, sometimes observed when using agonists (20, 21). In a review of the associated adverse side effects, the incidence of nausea, vomiting, and diarrhea was reasonably common (1 in 10) with ≤50% of patients affected by nausea (22), resulting in discontinuation rates of ≤10% in clinical practice (23). Secondly the vagal/neural dependency of part of GLP-1 action might not be accessible for GLP-1 enhancers/analogs (24, 25). For example, pharmacological GLP-1 administration may reduce appetite primarily via direct interaction with the brain (26), whereas endogenous GLP-1 effects on appetite might be exerted locally via receptors on the vagus nerve, and indirectly through gastric emptying (27, 28). Theoretically, continuous activation of the GLP-1 receptor via drug agonists could lead to receptor downregulation, and the natural rise and fall of nutrient-mediated GLP-1 secretion could be important for sustaining receptor sensitivity. Lastly, nutrient delivery might lead to increased secretion of colocalizing hormones including PYY, GIP, and oxyntomodulin (29) providing an additive effect on appetite suppression and blood glucose regulation.

The release of the incretin hormones, GLP-1 and GIP, and other gut hormones, including PYY and oxyntomodulin, is dependent on nutrient delivery to different enteroendocrine cells within the intestinal epithelium. Intestinal L-cells express several receptors and transporters selective to different macro and micronutrients (30). Because of this, whereas the extent of GLP-1 secretion is principally mediated by meal size (in order to reach the distal gut), the nutrient composition of a meal might also be influential for maximizing L-cell receptor activation and transporter utilization. Previously, carbohydrate, fat, and protein ingestion, as well as SCFAs produced by the gut microbiota, have been found to independently stimulate GLP-1 release (31–33). Emerging evidence suggests that the synergy of protein and calcium could also act as a potent secretagogue of GLP-1 (34, 35). In the most recent human study (35), this synergy produced some of the highest reported concentrations of GLP-1 following physiological ingestion to date. This is particularly interesting given that calcium does not provide energy, and therefore GLP-1 release is relatively greater despite no additional energy provision.

Previous reviews regarding the nutritional regulation of GLP-1 have discussed the specific molecular mechanisms underpinning fat-, carbohydrate-, and protein-mediated GLP-1 release (10, 36, 37). To our knowledge, only 1 review has addressed the effects of different nutrients on GLP-1 secretion (15); however, the effect of protein ingestion in humans was not discussed, as well as particular reference to protein forms and interaction with calcium, which is crucial for practical application. Therefore, this narrative review focuses on the incretin hormone GLP-1, and the research surrounding protein- and/or calcium-mediated GLP-1 secretion in humans. The potential mechanisms by which the combination of protein and calcium might enhance GLP-1 secretion are also discussed, alongside potential avenues for future research.

## Secretion and Metabolism of GLP-1

GLP-1 is a peptide hormone produced by intestinal L-cells by differential processing of the proglucagon (*GCG*) gene (38). GLP-1 exists in 2 equipotent molecular active forms, GLP-1_7–37_ and GLP-1_7–36amide_, with the later representing ∼100% of circulating GLP-1 in humans (39, 40). Following the release of active GLP-1, only ∼10–15% enters the systemic circulation due to rapid degradation by dipeptidyl peptidase-IV (DPP4) in the splanchnic bed (38). Further degradation is likely to occur in the circulation by soluble DPP4, including DPP4 released from adipose tissue (41, 42). DPP4 cleaves 2 N-terminal amino acids of GLP-1_7–37_ and GLP-1_7–36amide_ to produce GLP-1_9–37_ and GLP-1_9–36amide_, respectively, which act as low-affinity ligands for the GLP-1 receptor (43). Additionally, both the active and degraded forms of GLP-1 are cleared from the circulation via the kidneys (44). For the purpose of this review GLP-1_7–36amide_ will refer to the measurement of the receptor-active form of GLP-1, and GLP-1_TOTAL_ will incorporate GLP-1_7–36amide_ as well as the downstream metabolite, GLP-1_9–36amide_. Although plasma concentrations of GLP-1_7–36amide_ are the most convenient to sample, given such rapid degradation by DPP4, GLP-1_7–36amide_ concentrations recorded within lymph might be more sensitive to meal-induced GLP-1 secretion (45, 46). Lymphatic sampling is, however, currently challenging for human studies, and therefore plasma GLP-1 concentrations will be the main focus of this review.

GLP-1 secretion is regulated through meal intake, whereby GLP-1_TOTAL_ plasma concentrations are very low in the fasted state (5–15 pmol/L) and rise following the ingestion of food by 2- to 4-fold (15, 38, 47). GLP-1 release has been demonstrated to fit either a monophasic (48, 49) or biphasic pattern (50, 51), reflecting a gradual rise to peak, or an initial rapid rise ∼10–15 min postmeal, followed by a secondary steady rise to peak, respectively. Peak GLP-1 concentrations occur ∼45–60 min after a meal, before a gradual decrease until the next prandial episode (38, 50). The early GLP-1 response was thought to occur via neural activation involving the vagus nerve (31, 52) and/or direct stimulation of the proximal jejunum L-cells, with the majority of GLP-1 stimulation occurring through direct interaction with distal L-cells (38). However, more recently it has been recognized that the density of L-cells in the duodenum is enough to account for at least part, if not most, of the early response (53).

GLP-1 has several known actions including glucose-dependent stimulation of insulin release, and inhibition of glucagon secretion, gastric emptying, and food intake (38, 54, 55). These actions are coordinated via the GLP-1 receptor, a G-protein–coupled receptor widely expressed in the brain, heart, pancreatic islets, and the GI tract (38, 56). Despite these actions contributing to improved metabolic control, GLP-1 has a half-life of only ∼2 min (10). This suggests that its effects are not solely mediated through direct activation of local GLP-1 receptors but largely through paracrine receptor activation on vagal afferent nerve fibers innervating target organs (27, 38, 57). Consequently, nutritional interventions targeting enhanced and prolonged GLP-1 release are of high interest.

## Current Status of Knowledge

### Protein-mediated GLP-1 release

Protein is considered the most satiating of the macronutrients (58, 59), and is often associated with weight loss (60), which could be mediated in part by protein-induced stimulation of appetite regulatory hormones, including GLP-1. Traditionally, fat and carbohydrate were thought to be the most potent stimulants of GLP-1 release (61), but this has subsequently been challenged by research comparing protein ingestion with carbohydrate and fat in humans.

### Direct comparison between isocaloric macronutrients

A limited number of studies have compared GLP-1 release following ingestion of calorie- and/or volume-matched macronutrients. A comparison of the effect of high-protein compared with high-fat meals on GLP-1 release was studied in 12 healthy males who ingested milk and egg protein at 2 g/kg on one occasion and 0.88 g/kg oleic acid on the other (volume- and calorie-matched) (62). Following both meals, GLP-1_7–36amide_ and GLP-1_TOTAL_ increased proportionally but did not differ in magnitude between the 2 conditions. Another study compared GLP-1 release following the ingestion of whey protein with that of maltodextrin (45 g) in 18 healthy weight women (BMI 19–25 kg/m^2^) (63). GLP-1_7–36amide_ incremental area under the curve (iAUC) was not significantly different between conditions; however, there was a trend for greater GLP-1_7–36amide_ iAUC following the whey protein test meal. Furthermore, the acute effects of 3 isocaloric (375 kcal) test meals, high in carbohydrate (100 g glucose), fat (84 mL double cream), or protein (352 g grilled turkey), resulted in similar peak GLP-1_7–36amide_, although different times to peak (31). This might be explained by the different forms in which the 3 macronutrients were ingested, and the effect this could have on gastric emptying and subsequent delivery to the intestine. Although it is challenging to completely isolate one macronutrient from another, these studies provide some rationale to suggest that the ingestion of specific macronutrients largely results in comparable GLP-1 release. However, the measurement of GLP-1_TOTAL_ would have provided greater sensitivity to detect changes in GLP-1 secretion between conditions in some of these studies.

### Isocaloric meals high in protein, fat, or carbohydrate

A more applied approach would incorporate the interactions of different macronutrients, common to the typical diet. Ten healthy normal-weight and 10 overweight males were fed isocaloric pasta and dessert meals high in fat (65%), carbohydrate (66%), or protein (65%), with the remaining energy requirement shared equally between the other 2 macronutrients (∼66%:17%:17%). Satiety was greatest following the high-protein meal, despite no differences in GLP-1_7–36amide_ concentrations following each meal (64). Instead, this might be explained by the greater increase in PYY_3-36_ (the active form of a cosecreted L-cell–derived hormone) in the high-protein condition compared with the high-fat and high-carbohydrate conditions. In a similar study design, gut hormone responses of 8 healthy volunteers were measured following pancake breakfasts that provided 60% of energy from either protein, fat, or carbohydrate (65). The high-protein meal stimulated the greatest GLP-1_TOTAL_ and PYY_3-36_ secretion; however, this did not translate into substantial changes in subsequent food intake measured during an ad libitum lunch meal between conditions. Although, this might be due to the timing of the ad libitum lunch 4 h after the test meal, which coincided with a gradual rise of ghrelin concentrations in each condition. In order to assess the influence of relative protein dose on GLP-1 release, 25 overweight men were fed isocaloric test meals (adjusted at the expense of carbohydrate) of normal protein (14% energy from protein), medium-high protein (25%), and high protein (50%). A dose-dependent increase in mean GLP-1_7–36amide_ and PYY_3-36_ concentrations was recorded following meals with increasing protein intake (66). This dose-dependent increase in GLP-1 could have been due to greater protein delivery to more distal parts of the intestine with increasing protein load (38).

The short-term effects of high-protein meals on GLP-1 release and appetite seem to persist over longer time frames. For example, 12 healthy women were provided a high-protein diet (30% energy from protein, 40% carbohydrate, and 30% fat) compared with an adequate-protein diet (10% energy from protein, 60% carbohydrate, and 30% fat) for 4 d, in a randomized crossover design (67). GLP-1_7–36amide_ was measured for a 24-h period on day 4 of each respective diet. GLP-1_7–36amide_ was significantly greater 15 min following dinner, and tended to be greater following breakfast, after the high-protein compared with the adequate-protein diet. Accordingly, the 24-h AUCs for hunger and satiety were lower and greater, respectively, for the high-protein diet. These studies provide some evidence to suggest that, in both healthy and overweight individuals, a (mixed-macronutrient) diet with a high relative protein composition can enhance GLP-1 release to a greater extent than diets with a high relative proportion of carbohydrate or fat.

### Type and form of protein

Given that nutrient delivery and sensing are key mechanisms that regulate gut hormone secretion, it is important to consider the type and form of protein in the context of GLP-1 release.

#### Solid compared with liquid meals

It is well established that liquid meals empty the stomach at a faster rate than solid meals (68–70). Subsequently, faster nutrient delivery (following a liquid compared with a solid meal) could reduce the time window for intestinal absorption, thereby resulting in greater exposure of nutrients to more distal parts of the intestine, which could elevate GLP-1 release (38). Indeed, in 6 healthy lean males and females, GLP-1_7–36amide_ iAUC was significantly greater following a mixed macronutrient liquid compared with a solid meal, matched for energy content and volume (52% energy from carbohydrate, 34% energy from fat, 15% energy from protein) (71). Equally, GLP-1_7–36amide_ iAUC was elevated to a greater extent following a liquid mixed meal (345 kcal) compared with a solid mixed meal (362 kcal) in 6 participants who underwent recent surgical or medical weight loss within 1 y (72). Similar findings were also observed in a larger study incorporating 32 participants following RYGB surgery (73). GLP-1_TOTAL_ iAUC was greater following a mixed macronutrient liquid compared with a solid meal (matched for nutrient composition and energy content), although this occurred despite no differences in gastric pouch emptying time between liquid and solid meals. This suggests that other factors such as osmolarity could also be responsible for GLP-1 differences following solid and liquid meals (10, 74). Studies are required to compare GLP-1 release following the ingestion of matched protein meals in solid and liquid form to see whether effects of meal form are modulated by specific macronutrients.

#### Whey compared with casein

Whey and casein proteins are the major constituents of milk protein, representing 20% and 80% of milk protein, respectively. Previous research has provided inconclusive evidence about whether one protein is more satiating than the other; however, due to faster gastric emptying following whey compared with casein ingestion, whey has been considered more satiating in the short term (< ∼180 min), and casein more satiating in the long term (> ∼180 min) (75). Considering these differences, GLP-1 responses have been variable. Eight healthy females and 1 male ingested whey protein on one occasion and casein protein on another, in the form of a 48-g liquid preload (76). The whey protein preload resulted in a 65% greater GLP-1_7–36amide_ iAUC compared with casein over 90 min. Despite this, a longer measurement period might have been more appropriate considering the slower rate of GI transit following casein consumption. Following a longer measurement period of 180 min, there were no significant differences in mean postprandial GLP-1_7–36amide_ concentrations between whey and casein protein preloads (30 g) in 24 overweight/obese males and females (77). Moreover, to determine GLP-1 release independent of gastric emptying, 6 healthy males ingested whey and casein in both intact and hydrolyzed (partially digested) forms on separate occasions. There were no significant differences in mean postprandial GLP-1_7–36amide_ responses or gastric emptying half-times between conditions over 120 min (78).

#### Other protein sources

The ingestion of other sources of protein, including gluten, soy, and cod, has been shown to stimulate GLP-1 secretion, although the magnitude of these responses does not appear to differ substantially between source (79–81). This appears consistent in individuals with healthy normal weight (79), overweight/obesity (79, 80), and T2D (81). In summary, findings suggest that ingestion of liquid compared with solid mixed-meals (matched for nutrient composition and caloric content) results in greater GLP-1 secretion. Whey and casein, when emptied from the stomach at similar rates, have comparable GLP-1 responses. Finally, ingestion of other protein forms including soy and gluten results in similar GLP-1 responses to whey and casein in terms of magnitude. Isolating specific amino acids/peptides (available following protein digestion/absorption) could provide some indication as to what conditions are optimal for protein-mediated GLP-1 release.

#### Amino acids

Individual amino acids are capable of stimulating GLP-1 release, and glutamine (82, 83), phenylalanine (34, 84), arginine (85–87), and tryptophan (88) have been shown to be some of the most effective. In one study, different amino acids (all 10 mM) were perfused through isolated loops of rat small intestine to stimulate GLP-1 release (34). Results suggested that phenylalanine was the most potent amino acid to stimulate GLP-1 secretion, followed by arginine > glutamine ∼ tryptophan > asparagine. Conversely, 10 g l-phenylalanine did not enhance GLP-1 concentrations in comparison with placebo capsules in healthy humans (89). In another study, l-tryptophan (0.15 kcal/min), l-phenylalanine (0.45 kcal/min), and l-glutamine (0.45 kcal/min) were infused intraduodenally for 90 min before a buffet-style test meal in healthy normal weight men (90). The lower rate of infusion of l-tryptophan was due to poor tolerance of higher doses. GLP-1_TOTAL_ ΔiAUCs were comparable between amino acids, although food intake was reduced to a greater extent following l-tryptophan administration compared with l-phenylalanine and l-glutamine. More recently, each amino acid (apart from l-tyrosine due to solubility) was perfused into the lumen or vascular side of isolated rat small intestine (91). Luminal administration showed that l-valine, l-phenylalanine, and alanyl-l-glutamine (stable dipeptide isoform of l-glutamine) were the most powerful stimulators of GLP-1 secretion. Interestingly, vascular but not luminal administration of l-arginine and l-tryptophan resulted in 2.9- and 2.7-fold increases in GLP-1 secretion compared with baseline, which suggests that amino acid–mediated GLP-1 secretion occurs via absorptive and postabsorptive mechanisms (91). The potential to combine multiple amino acids/peptides to stimulate GLP-1 secretion is an exciting avenue for future nutritional interventions.

#### Summary

There is no conclusive evidence to suggest that proteins, fed in isolation, are more or less potent at stimulating GLP-1 secretion compared with other isolated macronutrients. However, the ingestion of mixed meals with high compared with low relative protein composition could be more effective at enhancing GLP-1 secretion. Protein feeding/administration alone is sufficient to enhance gut hormone release, and therefore identifying the most potent amino acids for stimulating gut hormone release could be beneficial for the design of protein forms/supplements to target enhanced gut hormone availability.

### Calcium effects on appetite and GLP-1 concentrations

Calcium intake has been inversely associated with BMI and body fat content (92–94). Moreover, chronic calcium supplementation has also been associated with weight loss (95, 96). Possible mechanisms that could be responsible for this include a reduction in fat absorption (97), increased fat utilization (98), and/or a direct calcium effect on appetite (99, 100). However, the difference in fat absorption reported by Christensen et al. (97) between calcium and control supplements (increase in 1.6–8.8 g/d fecal fat) relates to 15.6–86 kcal/d and therefore is probably not meaningful for energy balance (101). Furthermore, subsequent research has found no evidence of calcium supplementation enhancing fat utilization, at rest or during physical activity (102, 103).

The effect of calcium on appetite could be related to its influence on appetite regulatory hormones. Following the ingestion of a high-calcium compared with a low-calcium mixed macronutrient breakfast there was a 22% increase in GLP-1_7–36amide_ iAUC over 120 min in healthy weight individuals (103). This was mirrored by a 19% increase in insulin iAUC for the high-calcium compared with the low-calcium condition. Conversely, in healthy overweight individuals, no differences in postprandial gut hormone concentrations were reported over 420 min in response to isocaloric low-, medium-, or high-calcium meals, or calcium carbonate supplement (104). There was, however, a lower triglyceride response in the medium- and high-calcium conditions compared with the calcium supplement, potentially suggesting decreased fat absorption. Nevertheless, there were no differences in appetite sensation between conditions.

Chronic calcium supplementation might also enhance GLP-1 availability. In a randomized crossover design, participants were provided with a defined diet including bread supplemented with 1 g calcium phosphate per day on one occasion and a placebo bread on the other (separated by a 2-wk washout) (105). GLP-1 responses were measured following a single administration and following the 3-wk intervention. No differences occurred following the single administration between conditions; however, plasma GLP-1_TOTAL_ and GLP-1_7–36amide_ AUC were significantly greater following the 3-wk intervention with calcium phosphate compared with the 3-wk placebo (105).

With a relatively small body of literature and different study designs, more research is required to determine whether isolated calcium ingestion can specifically enhance GLP-1 availability, or whether calcium is in fact reliant on the co-ingestion of other macronutrients, including protein.

### Protein and calcium co-ingestion enhances GLP-1 release

Foods containing both protein and calcium, for example, dairy, have been shown to stimulate GLP-1 release. Forty-nine overweight men and women were randomly assigned to either a high-dairy diet (∼1400 mg/d dairy) or a control diet (∼700 mg/d dairy) for 12 wk (106). The change in GLP-1_7–36amide_ concentrations between baseline and week 12 was significantly greater in the high-dairy condition, although this did not translate into any differences in weight loss between the 2 groups. More recently, gut peptide responses to cheese were measured using a murine intestinal STC-1 cell line (107). Nine of 10 water-soluble extracts of Irish cheddar cheeses stimulated GLP-1 secretion compared with the vehicle control, and all 10 cheeses significantly inhibited DPP4 compared with the buffer alone. In addition, 12 healthy participants were served test meals of reconstituted milk, cheese, whey protein, cod, and wheat gluten (108). There were no differences in GLP-1_TOTAL_ AUC over 60 min between any conditions. A longer measurement period with greater resolution might, however, have been necessary for measuring the complete GLP-1 response.

**FIGURE 1:**
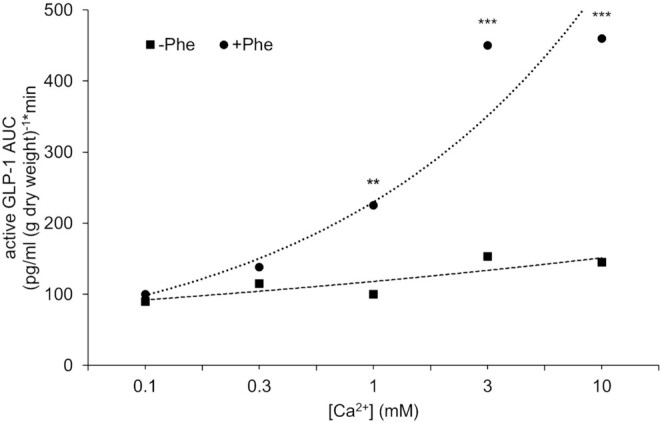
GLP-1 release in response to phenylalanine and accumulative calcium perfusion. Rat small intestine was perfused with Krebs–Henseleit buffer deplete of calcium ± 10 mM phenylalanine. At 20 min, Ca^2+^ was reintroduced cumulatively into the perfusate every 15 min over 90 min. The figure depicts the extracellular Ca^2+^–GLP-1 response relation using AUC for each Ca^2+^ addition. Student unpaired *t* tests were used to determine significance between control and phenylalanine: **P < 0.01, ***P < 0.001. GLP-1, glucagon-like peptide 1. Reproduced from reference 34 with permission.

Although these aforementioned studies utilized meals high in calcium and protein, further research has specifically isolated protein and calcium and demonstrated a potent synergy on GLP-1 release. The first of these studies, previously alluded to, used loops of rat small intestine to perfuse different amino acids in the absence or presence of calcium (34). In the presence of extracellular Ca^2+^, GLP-1 was secreted following the perfusion of each amino acid. However, the absence of extracellular Ca^2+^ during perfusion of amino acids completely abolished GLP-1 secretion. Phenylalanine was reported to be the most potent amino acid for stimulating GLP-1 release and was therefore perfused for 90 min in a Ca^2+^-deplete buffer. Ca^2+^ concentration was then progressively increased in 0.1, 0.3, 1, 3, to 10 mM steps every 15 min. Phenylalanine-mediated GLP-1 release was augmented by increasing Ca^2+^ concentrations (**Figure 1**). This phenylalanine-calcium synergy was also apparent for the release of PYY and GIP.

The effect of this synergy on gut hormone release was subsequently explored in humans. Following an overnight fast and habitual diet and activity standardization, participants were fed either a control (CON, ∼104 mg calcium and 4 g protein), high-protein (PRO, ∼104 mg calcium and 29 g protein), high-calcium (CAL, ∼1170 mg calcium and 5 g protein), or a high-protein and high-calcium (PROCAL, ∼1170 mg calcium and 29 g protein) preload (109). Blood samples were collected every 15 min for 1 h. Whereas GLP-1_7–36amide_ iAUC was higher following PRO and PROCAL compared with CON, there were no clear differences between PRO and PROCAL. However, it could be that the collection period was not long enough to capture the entire response, or that the measurement of venous, as opposed to arterialized, GLP-1_7–36amide_ concentrations was problematic for detecting differences between conditions in terms of rate of secretion (110). Interestingly, ad libitum energy intake was overcompensated for with CAL and PROCAL compared with PRO, suggesting these conditions suppressed appetite to a greater extent than protein alone.

In a series of acute experiments, Chen et al. (35) provided further evidence of a synergy between calcium and protein. In the first study, preloads (matched for calcium content—1000 mg) consisted of 4380 mg calcium citrate, 3745 mg milk minerals high in calcium, and 2050 mg milk minerals high in calcium with 50 g whey protein hydrolysate. Milk minerals and protein increased GLP-1_TOTAL_ iAUC by 9-fold compared with calcium citrate and milk minerals alone. The second study aimed to assess whether the addition of milk minerals rich in calcium to whey protein hydrolysate enhanced GLP-1 secretion compared with whey protein hydrolysate alone. Plasma GLP-1_TOTAL_ iAUC was ∼25% greater following milk minerals and protein ingestion compared with protein alone, although no differences were reported for GIP and PYY iAUC. Importantly, the GLP-1_TOTAL_ concentrations measured in response to the co-ingestion of protein and milk minerals were some of the highest ever reported following meal ingestion in humans. Peak plasma GLP-1_TOTAL_ concentrations reached 91 ± 20 pmol/L for milk minerals and protein, compared with 43 ± 12 pmol/L and 78 ± 20 pmol/L for milk minerals alone and protein conditions alone, respectively.

#### Summary

Animal models and human studies have provided initial evidence of a potent synergy between protein and calcium on GLP-1 secretion. However, understanding the mechanisms behind this synergy are fundamental for optimizing nutrition to maximize GLP-1 availability.

### Putative mechanisms underpinning potential protein- and calcium-mediated GLP-1 release

Nutrients that pass through the intestinal lumen are sensed by open-type enteroendocrine cells, which in turn leads to the release of peptide hormones from secretory granules into the lamina propria, before being taken up by blood capillaries or lymphatic vessels (38, 111, 112). Membrane-bound receptors and transporters are therefore pivotal for the detection and absorption of these nutrients, and many macronutrient-specific receptors have been distinguished. Notable receptors/transporters involved in amino acid sensing include: G-protein–coupled receptor class C 6A (GPRC6A) (113), G-protein–coupled receptor 142 (114), type 1 taste receptors (T1Rs) (115), and sodium-dependent neutral amino acid transporter (B AT1) (30). Receptors for peptides/oligopeptides include: G-protein–coupled receptor 93 (GPR93) (116) and peptide transporter 1 (PEPT1) (117). Additionally, the extracellular calcium-sensing receptor (CaSR) is receptive to both amino acids and peptides (30, 118, 119). Amongst these, CaSR, GPRC6A, and PEPT1 are all reportedly selective/sensitive to calcium.

### The CaSR is present in the GI tract and responds to extracellular amino acids and calcium

CaSR is a class C G-protein–coupled receptor (GPCR) that responds to calcium as its principal physiological ligand (120, 121). However, some agonists are able to modify the receptor's affinity via allosteric actions (type 2 agonists). Amino acids are type 2 agonists that can bind to the extracellular domain of the CaSR and modulate its activity (122). Furthermore, the CaSR binds extracellular Ca^2+^ over a concentration range of 0.5–10 mM (122). Therefore, in the presence of these concentrations of calcium, CaSR can act as an l-amino acid receptor. Importantly, CaSR operates in a reciprocal fashion. While being directly activated by Ca^2+^, occupancy of the l-amino acid binding site enhances the sensitivity of CaSR to calcium (120, 123). Therefore, during a meal in which calcium and protein are in abundance, calcium and amino acids could act as coagonists of the CaSR (120). However, the CaSR is not responsive to every amino acid. Aromatic amino acids, including phenylalanine and tryptophan, and aliphatic amino acids are the most efficient agonists of CaSR. Charged amino acids are significantly less effective, with branched-chain amino acids almost ineffective (120).

The CaSR has been localized through immunostaining on both the apical and basolateral membranes of the small and large intestine villus and surface cells in both humans (124) and rats (125, 126). However, evidence appears inconclusive as to which site is most capable of sensing amino acids and initiating GLP-1 release. Using isolated perfused rat small intestine, calindol, an allosteric modulator of CaSR, strongly stimulated GLP-1 release when infused intravascularly but had minimal effects when administered intraluminally (127). Additionally, recent work utilizing the same model demonstrated that vascular inhibition of CaSR with NPS2143 (a CaSR antagonist) significantly reduced amino acid–stimulated GLP-1 release (91). This suggests that amino acid/peptide absorption is important, whereby amino acid delivery to the vasculature through basolateral transporters then activates CaSR located on the basolateral membrane (91, 127). Despite this, other reports suggest that CaSR can sense luminal nutrients (122), and that GLP-1, unlike GIP, does not necessarily require intestinal absorption for it to be stimulated (128).

The CaSR in particular, appears to be an important receptor for calcium- and protein-mediated GLP-1 release. Calhex 231 (another CaSR inhibitor) significantly inhibited amino acid–induced stimulation of GLP-1 by ∼70% in the presence of extracellular Ca^2+^, using isolated loops of rat small intestine (34). Equally, Calhex 231 and NPS2143 both significantly lowered peptone-triggered GLP-1 secretion from primary colonic cultures (117). Furthermore, unlike many other receptors, CaSR undergoes agonist-driven insertional signaling, making it highly resistant to functional desensitization (118, 129, 130) and therefore potentially capable of sustaining prolonged GLP-1 release in response to amino acids and calcium.

### GPRC6A contains a calcium-binding site and could play a minor role in protein-and calcium-mediated GLP-1 release

GPRC6A is also a class C GPCR that shares 32% amino acid identity with CaSR, suggesting they could share significant functional properties (131, 132). GPRC6A is reportedly activated by a variety of ligands: osteocalcin, testosterone, basic amino acids, and divalent and trivalent cations (133, 134). Additionally, GPRC6A possesses a calcium-binding site, albeit with a weaker affinity than CaSR (135), although it is thought that this binding site allows direct activation of GPRC6A by calcium (136). When cells expressing GPRC6A were preincubated in physiological saline solution of low Ca^2+^ concentration (0.5 mM) and then switched to high Ca^2+^ (5 mM), several amino acids demonstrated intracellular Ca^2+^-mobilizing responses that were not evident under control conditions (137). The enhanced Ca^2+^-mobilizing responses could theoretically lead to greater GLP-1 exocytosis.

GPRC6A is activated by basic amino acids, which trigger GLP-1 secretion in GLUTag cell lines (derived from colonic tumors of transgenic mice expressing T antigen), but not in primary murine intestinal cultures (113, 134). For instance, l-ornithine administration significantly and dose-dependently increased intracellular Ca^2+^ concentrations, which correlated with increased GLP-1 secretion in a GLUTag cell line compared with unstimulated control cells (113). However, in the presence of a GPRC6A antagonist, calindol, l-ornithine–induced calcium elevations and subsequent GLP-1 release were suppressed. The authors also transfected GPRC6A small interfering RNAs into GLUTag cells and successfully reduced the expression of GPRC6A. Accordingly l-ornithine–induced GLP-1 release was significantly decreased. Despite this, utilizing a rodent GPRC6A whole-body knockout model, oral administration of both l-arginine and l-ornithine significantly increased plasma GLP-1_TOTAL_ to a comparable magnitude in GPRC6A knockout and wild-type mice (138). However, as extensively discussed by Pi et al. (133, 139), caution should be taken when analyzing the reported effects. GPRC6A mouse knockout models are challenging due to variations in metabolic phenotype (140, 141), which are reportedly influenced by environmental challenges and genetic differences in mouse strains (139). In addition, the translational validity of such studies is questioned because GPRC6A (in humans) presents an evolutionarily divergent genotype characterized by a gain-of-function polymorphism (GPRC6A^KGKY^), which might alter the role of this receptor (133). The research so far suggests that GPRC6A likely plays a role in the coordination of GLP-1 release but is not essential for it. Further studies in humans are necessary to fully understand the contribution of this receptor to GLP-1 secretion and metabolic health.

### GPCR signaling involves 2 major pathways leading to GLP-1 exocytosis

**FIGURE 2:**
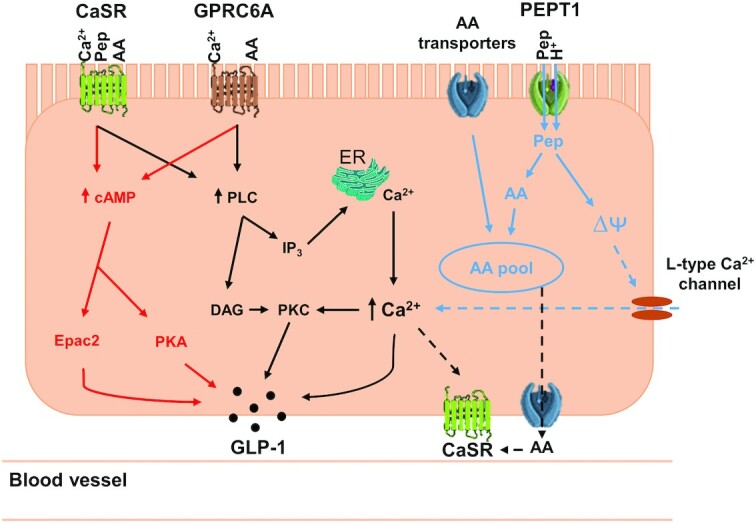
The potential putative mechanisms of calcium and protein synergy inducing GLP-1 secretion. Amino acids and peptides are sensed by CaSR (amino acids and peptides) and GPRC6A (amino acids only). The ability of CaSR to bind both peptides and amino acids is dependent on the presence of calcium, whereas GPRC6A also contains a calcium-binding site. This binding leads to 2 downstream signaling pathways involving cAMP (red) and phosphatidylinositol (black), leading to GLP-1 exocytosis. PEPT1 mediates the transport of peptides into the cell and subsequently causes membrane depolarization (potentially leading to calcium influx via L-type Ca^2+^ channels—blue dashed arrows) and ultimately GLP-1 exocytosis. Amino acids that enter the cell via amino acid transporters, including B˚AT1, and peptides that are broken down, join the amino acid intracellular pool. Intracellular amino acids and peptides transported out of the cell might also be sensed by CaSR localized on the basolateral membrane (black dashed arrows), which similarly triggers the signaling pathways highlighted in red and black. AA, amino acids; B˚AT1, sodium-dependent neutral amino acid transporter; CaSR, extracellular calcium-sensing receptor; DAG, diacylglycerol; Epac2, exchange protein directly activated by cAMP 2; ER, endoplasmic reticulum; GLP-1, glucagon-like peptide 1; GPRC6A, G-protein–coupled receptor class C 6A; IP_3_, inositol 1,4,5-triphosphate; Pep, peptides; PEPT1, peptide transporter 1; PKA, protein kinase A; PKC, protein kinase C; PLC, phospholipase C; ∆ᴪ, membrane depolarization.

GPCRs, including CaSR and GPRC6A, represent the largest group of cell surface receptors. Following receptor binding, intracellular G-proteins are activated and couple to 2 main signaling pathways: the cAMP pathway and the phosphatidylinositol pathway (130, 142), illustrated in **Figure 2**. The former involves the modulation of cAMP, which activates downstream targets Epac2 (exchange protein directly activated by cAMP 2) and protein kinase A, influential for GLP-1 exocytosis (142–144). The latter pathway involves the activation of phospholipase C, which in turn mediates the production of diacylglycerol and inositol 1,4,5-triphosphate. These lead to the activation of protein kinase C and mobilization of Ca^2+^ from the endoplasmic reticulum, respectively, leading to GLP-1 exocytosis (142, 145, 146).

### Peptide transporter 1 provides intestinal transport for di-/tripeptides and might involve voltage-gated Ca^2+^ channel opening

The oligopeptide transporter, peptide transporter 1 (PEPT1), is located in the intestinal brush border membrane and has an important role in protein absorption in the intestine (147). PEPT1 is selective for dipeptides and tripeptides, but not for free amino acids or peptides containing ≥4 amino acids (148). PEPT1 is a member of the Slc15 family of proton co-transporters and its unique feature of using an inwardly directed proton gradient enables peptides to enter the cell even against a concentration gradient (148). This leads to membrane depolarization, subsequent calcium entry, and enteroendocrine hormone secretion (142). PEPT1-mediated transport of dipeptides has been shown to elicit hormone secretion in PEPT1-transfected STC-1 cells (149). The authors used human growth hormone (GH) co-transfection with PEPT1 as a reporter for PEPT1-mediated hormone secretion. They reported that 10 mM glycine-glycine (Gly-Gly) and 10 mM glycine-sarcosine (Gly-Sar) increased GH secretion in PEPT1-transfected STC-1 cells 3.8- and 4-fold, respectively. Moreover, using primary cultures from murine colon, the PEPT1 substrate cefalexin enhanced GLP-1 secretion 1.4-fold above baseline, suggesting a key coupling between PEPT1 and L-cell activation (117). In support, the GLP-1 secretory response to the non-hydrolysable Gly-Sar was suppressed by the PEPT1 antagonist 4-AMBA (117). Gly-Sar–mediated GLP-1 secretion was also sensitive to calcium, whereby Gly-Sar–induced GLP-1 secretion was completely inhibited in the absence of extracellular calcium or by the L-type calcium channel blocker nifedipine. Equally, peptone-mediated GLP-1 secretion was inhibited in a rat intestine perfusion model by 4-AMBA (127). However, amino acid–mediated GLP-1 secretion was also suppressed by 4-AMBA, suggesting the observed antagonism might not be fully specific for PEPT1. In contrast to the previously mentioned study, opening of L-type Ca^2+^ channels was not essential for peptone-mediated GLP-1 secretion, because nifedipine only blocked GLP-1 secretion following the luminal perfusion of glucose and not peptones (127). These findings suggest that calcium influx could be important for PEPT1-mediated hormone secretion in L-cells. Absorption of di-/tri-peptides into the cell could also be important for the basolateral CaSR sensing of amino acids/peptides when they are transported out of the cell.

#### Summary

The speculated mechanisms behind calcium- and protein-mediated GLP-1 release are summarized in Figure 2. CaSR is the key component of this working model, where both CaSR and GPRC6A receptors demonstrate a key coupling of amino acids/protein and calcium to trigger GLP-1 release. The potential localization of CaSR on both the luminal and vascular membrane of the L-cell could mediate GLP-1 release through luminal sensing of peptides and amino acids, and also basolateral sensing of amino acids in the vasculature. Basolateral sensing relies on luminal absorption of di- and tri-peptides/amino acids into the cell through PEPT1 and amino acid transporters including B^o^AT1, and then autocrine action following movement of amino acids out of the cell via basolateral transporters.

## Areas for Future Research

As highlighted in this narrative review there is a limited number of human studies that have investigated the effect of protein and calcium co-ingestion on the secretion of endogenous GLP-1, and further studies are required to support the encouraging findings reported so far (35). Whereas many studies have investigated the effects of manipulating the type and amount of protein on gut hormone responses, less is known about the optimal dose of calcium to maximize protein-stimulated GLP-1 release. Although almost all dietary calcium is absorbed from the upper intestine (150), it might be possible that calcium can reach more distal areas of the intestine following a large dose. This could lead to increased GPCR activation and calcium absorption into the intracellular Ca^2+^ pool, and subsequently greater exocytosis of GLP-1. Further studies are also required to confirm the mechanisms speculated in the previous section. In particular, it is important to determine whether basolateral or apical localization of CaSR is more significant for gut hormone release, or whether they are equally vital. This would provide information on whether CaSR-mediated GLP-1 secretion is more dependent on nutrient contact, nutrient absorption, or both.

Currently, the only research conducted in humans using a protein-calcium design has sampled healthy, lean participants and is acute in nature (35, 109). Studies in individuals with overweight/obesity and T2D are required to determine if this effect is present in clinical populations requiring nutritional interventions for the regulation of blood glucose and weight loss. It is also important to determine whether this heightened response can be sustained chronically, potentially by an intervention diet high in protein and calcium, or by manipulating certain meals of the day, for example, breakfast, to feed a high-protein/high-calcium drink/supplement. Chronically, it might also be possible to influence the enteroendocrine cell population via nutritional intervention considering the rapid turnover of enteroendocrine cells of ∼5 d (151). If a high-protein/high-calcium meal intervention could upregulate intestinal L-cell growth, or equally the abundance of GPCRs at the cell membrane, this could be hugely important for gut hormone availability and associated blood glucose and appetite regulation.

Lastly, given that a number of receptors are selective to a range of different amino acids and peptides, the design of certain amino acid/peptide mixtures to maximize multiple receptor stimulation should be tested in vitro, and then, following optimization, in human participants. This could lead to the possibility of evoking a substantial GLP-1 response without the need to feed calorie-dense meals. This could improve weight loss via 2 main mechanisms: *1*) by suppressing hunger through a potent peptide hormone response; and *2*) the low-caloric nature of the nutrient stimulation.

## Conclusion

Emerging research provides evidence of a potential synergy between protein and calcium on GLP-1 secretion. This synergistic effect has been substantiated by findings in cell cultures, animal models, and in human studies. The collective activation of different receptors/transporters as highlighted in our working model provides some insight into how this synergy might work on a mechanistic level. Future studies are required to substantiate this model and provide evidence of elevated GLP-1 responses translating to appetite suppression and blood glucose control, given the great potential to provide this nutritional stimulus in low-caloric doses.

## Notes

JDW is supported by a PhD studentship from Arla Foods Ingredients (DK). FK thanks MRC (UK) for funding (MR/P002927/1). JTG has received research funding from Arla Foods Ingredients (DK), LucozadeRibena Suntory Ltd (UK), and Kenniscentrum Suiker & Voeding (NL); and has acted as a consultant to PepsiCo (UK). These funders had no role in the design, implementation, analysis, or interpretation of this manuscript.

Author disclosures: The authors report no conflicts of interest.

Abbreviations used: B˚AT1, sodium-dependent neutral amino acid transporter; CaSR, extracellular calcium-sensing receptor; DPP4, dipeptidyl peptidase-IV; GH, growth hormone; GI, gastrointestinal; GIP, glucose-dependent insulinotropic polypeptide; GLP-1, glucagon-like peptide 1; GPCR, G-protein–coupled receptor; GPRC6A, G-protein–coupled receptor class C 6A; iAUC, incremental area under the curve; PEPT1, peptide transporter 1; PYY, peptide tyrosine-tyrosine; RYGB, Roux-en-Y gastric bypass surgery; Sar, sarcosine; T1R, type 1 taste receptor; T2D, type 2 diabetes.
